# How long can nosocomial pathogens survive on textiles? A systematic review

**DOI:** 10.3205/dgkh000345

**Published:** 2020-05-15

**Authors:** Günter Kampf

**Affiliations:** 1 University Medicine Greifswald, Institute for Hygiene and Environmental Medicine, Greifswald, Germany

**Keywords:** survival, pathogens, textiles, fabrics

## Abstract

**Aims:** Healthcare-associated infections linked to contaminated textiles are rare but underline their potential role as a source for transmission. The aim of the review was to summarize the experimental evidence on the survival and persistence of the different types of nosocomial pathogens on textiles.

**Methods:** A literature search was performed on MedLine. Original data on the survival of bacteria, mycobacteria, and fungi and persistence of viruses on textiles were evaluated.

**Results:** The survival of bacteria at room temperature was the longest on polyester (up to 206 days), whereas it was up to 90 days for some species on cotton and mixed fibers. Only low inocula of 100 CFU were found on all types of textiles with a short survival time of ≤3 days. Most bacterial species survived better at elevated air humidity. The infectivity of viruses on textiles is lost much faster at room temperature, typically within 2–4 weeks.

**Conclusions:** Contaminated textiles or fabrics may be a source of transmission for weeks. The presence of pathogens on the coats of healthcare workers is associated with the presence of pathogens on their hands, demonstrating the relevance of textile contamination in patient care.

## Zusammenfassung

**Zielsetzung:** Nosokomiale Infektionen, die von kontaminierten Textilien ausgehen, sind selten, zeigen aber dennoch ihr Potenzial als Quelle einer Übertragung. Ziel der Übersichtsarbeit war es, experimentell gewonnene Daten zum Überleben bzw. zur Persistenz verschiedener nosokomialer Pathogene auf Textilien zusammenfassend darzustellen.

**Methode:** Eine Literaturrecherche wurde auf MedLine durchgeführt. Originaldaten zum Überleben von Bakterien, Mykobakterien und Pilzen sowie zur Persistenz von Viren auf Textilien wurden ausgewertet.

**Ergebnisse:** Das Überleben von Bakterien bei Raumtemperatur war am längsten auf Polyester (bis zu 206 Tage), wohingegen es auf Baumwollen bzw. gemischten Fasern bis zu 90 Tage betrug. Nur kleine Inokula von 100 KBE konnten auf allen Textilien über maximal 3 Tage überleben. Die Mehrzahl der Bakterienspezies überlebte bei höherer Luftfeuchtigkeit besser. Die Infektiosität der Viren ging auf Textilien bei Raumtemperatur schneller verloren, meist innerhalb von 2–4 Wochen.

**Fazit:** Kontaminierte Textilien können über Wochen eine Quelle für Übertragungen darstellen. Der Nachweis von Pathogenen auf den Kitteln der Mitarbeiter war mit dem Nachweis der Pathogene auf ihren Händen assoziiert. Auch darin zeigt sich die Bedeutung der Kontamination von Textilien in der Patientenversorgung.

## Introduction

Healthcare-associated infections linked to contaminated textiles are rare, but play a role as a potential source of transmission. One example is the spread of group A streptococcus infections. An outbreak on a geriatric medical ward was explained by the presence of one healthcare worker (HCW) on the ward who was perineal carrier. Contamination of a fabric-upholstered chair used by the HCW in an office adjacent to the ward was also detected and was suspected to have enhanced the transmission to other HCWs [1]. Another example is an outbreak of meropenem-resistent *A. baumannii* on an intensive care unit. The major source appeared to be the curtains surrounding the patient’ beds [2]. Feather pillows have been described as an unexpected source of *Acinetobacter* spp., potentially causing outbreaks [3]. Outbreaks caused by bacterial spores on linen have also been reported, e.g., resulting in bacteraemia [4], [5]. Work garments have also been described to be contaminated with various types of microorganisms. The cuffs of long-sleeved coats frequently contact patients or environmental surfaces [6]. Soiled linen was also described as the source of tinea corporis infections in two HCWs who had only indirect contact to a patient infected with *T. tonsurans* [7]. The survival of nosocomial pathogens on inanimate surfaces has been well described [8]. But the persistence of pathogens on different types of textiles has not been reviewed. The purpose of this review was therefore to summarize the experimental evidence on the survival and persistence of the different types of nosocomial pathogens on textiles.

## Methods

A MedLine search was performed on the 29^th^ and 31^st^ of May, 2019. The following terms were used: cotton bacteria survival (361 hits), cotton virus survival (155 hits), cotton virus persistence (39 hits), cotton yeast survival (16 hits), cotton fungus survival (267 hits), cotton mycobacterium survival (19 hits), polyester bacteria survival (327 hits), polyester virus survival (57 hits), polyester virus persistence (8 hits), polyester yeast survival (13 hits), polyester fungus survival (174 hits), polyester mycobacterium survival (13 hits), wool bacteria survival (46 hits), wool virus survival (26 hits), wool virus persistence (8 hits), wool yeast survival (5 hits), wool fungus survival (31 hits), wool mycobacterium survival (5 hits), silk bacteria survival (51 hits), silk virus survival (14 hits), silk virus persistence (0 hits), silk yeast survival (6 hits), silk fungus survival (32 hits), and silk mycobacterium survival (0 hits). Publications were included and results were extracted from them when they provided original data on the survival or duration of persistence of bacteria, mycobacteria, fungi or viruses on textiles. Articles were excluded when they did not provide any original data on survival or persistence. Reviews were also excluded, but screened for any information within the scope of the review.

## Results

### Bacteria

#### Cotton

On cotton, many bacterial species are able to survive at room temperature for long periods of time, such as *Enterococcus* spp. (up to 90 d), *P. aeruginosa* (up to 8 w), *S. aureus* (up to 8 w), *K. pneumoniae* (up to 8 w), *S. pyogenes* (up to 46 d), *E. coli* (up to 45 d), *Enterobacter* spp. (up to 35 d), *S. sonnei* (up to 27 d), coagulase-negative *Staphylococcus* spp. (up to 27 d), *Acinetobacter* spp. (up to 25 d), *P. mirabilis* (up to 9 d) and *S. maltophilia* (up to 7 d). Other species at a high initial cell count, however, survive only for short periods of time at room temperature, e.g., *N. gonorrhoeae* and *S. marcescens* (both up to 3 d), *B. fragilis*, *B. cepacia* and C*. diphtheriae* (all up to 2 d), *P. vulgaris* (up to 1 d), *V. cholerae* (up to 8 h), *Salmonella* spp. (up to 5 h), *C. jejuni* (up to 3 h) and *F. nucleatum* (up to 2 h). At lower temperatures, the survival may be longer, as shown for *S. sonnei *and *S. equi*. A low inoculum of approximately 100 CFU was found for many species with only a short survival period of 2 h *(Acinetobacter* spp.) or ≤1 h (*E. coli*, *P. mirabilis*, *P. aeruginosa* and *S. marcescens*). Only *Enterobacter* spp. was able to survive much longer (up to 3 d) when inoculated with only 100 CFU. *M. bovis* survived on cotton for 2 m. Higher air humidity was associated with longer survival of *E. coli*, whereas lower air humidity enhanced the survival of *S. aureus* and *S. pyogenes* (Table 1 (Tab. 1)).

#### Synthetic fibers

On synthetic fibers such as polyester, the survival times of high inocula at room temperature ranged from up to 206 d (*E. coli*, *S. aureus*, *S. pyogenes*), 90 d *(Enterococcus* spp.), 56 d (*K. pneumoniae*, *P. aeruginosa*), 26 d (*Enterobacter* spp.), 14 d *(Acinetobacter* spp.) to 7 d (*S. marcescens*). A low inoculum of approximately 100 CFU was found for many species with only a short survival period of 2 d (*K. pneumoniae*), 1 d *(Enterobacter* spp.), 8 h (*Acinetobacter* spp.), 2 h (*P. mirabilis*) or ≤1 h (*E. coli*, *P. aeruginosa*, *S. marcescens*). Higher air humidity favored longer survival of *E. coli*, *S. aureus* and *S. pyogenes* (Table 2 (Tab. 2)).

#### Mixed and other fibers

High inocula applied to mixed and other fibers were able to survive at room temperature for up to 90 d *(Enterococcus* spp.), 49 d *(Enterobacter* spp.), 45 d (*E. coli*), 41 d (*S. aureus*), 33 d (*P. aeruginosa*), 28 d (coagulase-negative *Staphylococcus* spp.), 19 d *(Acinetobacter* spp.), 14 d (*K. pneumoniae*, *M. morganii*, *P. mirabilis*), and 7 d (*S. maltophilia*). Short survival times were found with *S. typhimurium* (≥1 d), *N. gonorrhoeae* (up to 1 d), and *S. dysenteriae* (4 h). Low inocula of 100 CFU were often associated with shorter survival times, e.g., in *K. pneumoniae* (1–3 d), *Acinetobacter* spp. (7 h), *E. coli* (≤6 h) or *P. mirabilis*, *P. aeruginosa* and *S. marcescens* (≤1 h). Longer survival was associated with higher air humidity in *K. pneumoniae*, *M. morganii*, *P. mirabilis*, *S. aureus* and *S. epidermidis* (Table 3 (Tab. 3)).

### Fungi

Most fungal species applied as high inocula were able to survive at room temperature on various types of fibers for 30 d or more (*A. fumigatus*, *C. glabrata*, *C. krusei*, *C. parapsilosis*, *C. tropicalis*, *C. neoformans*), 21 d (*G. candidum*), or 14 d (*C. albicans*). The differences between the fiber materials were variable. Four fungal species survived better on cotton or wool, three species on the blended fiber, and two on silk (Table 4 (Tab. 4)).

### Viruses

#### Cotton

At room temperature, some viruses persisted for long periods of time, such as the variola virus (18 m), vacciniavirus (up to 2 w), rabbit haemorrhagic disease virus (up to 10 d) or poliovirus (up to 7 d). The majority of viruses lose their infectivity at room temperature on cotton within 1 d (coronavirus, cytomegalievirus, ebolavirus, influenza A virus, influenza B virus, metapneumovirus). Low inocula have a substantially shorter persistence, as demonstrated with the coronavirus. The variola virus remained infectious for more than 10 years at 4°C (Table 5 (Tab. 5)).

#### Synthetic fibers

On synthetic fibers, some viruses kept their infectivity at room temperature for 12 d (ebolavirus), up to 7 d (influenza A virus, norovirus), or up to 3 d (calicivirus). The metapneumovirus could only persist for less than 1 d (Table 6 (Tab. 6)).

#### Mixed and other fibers

On the different types of fibers, viruses kept their infectivity at room temperature for up to 28 d (vacciniavirus), 14 d (calicivirus, norovirus), 12 d (foot-and-mouth disease virus) and 10 d (poliovirus). The influenza A virus, however, persisted only for up to 1 h. A low temperature enabled the vacciniavirus to persist longer, whereas the foot-and-mouth disease virus lost its infectivity sooner (Table 7 (Tab. 7)).

## Discussion

The compilation of data shows that the survival of bacteria at room temperature was the longest on polyester (up to 206 d), whereas it was 90 d for some species on cotton and mixed fibers. Only low inocula of 100 CFU were found on all types of textiles with a short survival time of ≤3 d. Most bacterial species survived better at elevated air humidity. The infectivity of viruses on textiles is lost much faster at room temperature, typically within 2–4 w. These data show that contaminated textiles may well serve as a source of transmission, provided the inoculum is high enough. Elevated air humidity is an advantage for survival of bacteria.

These data may have implications for the washing intervals of clothes worn at work. The duration of wear has an impact on the overall microbial load. It has been shown that the bacterial contamination of nurses’ coats is significantly higher after the second shift than after the first [9]. The change intervals in clinical practice may not reflect the real risk of contaminated clothes. In France, doctors changed their coats on average every 20 days [10]. Contaminated clothes may also have an impact on the contamination of the HCWs’ hands and vice versa. The presence of pathogens on coats is associated with the presence of pathogens on the hands of HCWs, whence they were probably originally transferred to the coats. Nevertheless, this still suggests that a contaminated coat can serve as a reservoir for contamination of the HCWs’ hands [11]. It has therefore been proposed that doctors leave their arms bare below the elbows, hang up their coat before patient contact, and launder their coat daily [12].

The impregnation of textiles with antimicrobial agents such as silver compounds, triclosan or copper has also been discussed to reduce their contamination in healthcare [13]. Copper-impregnated textiles can reduce multi-resistant bacterial species within 1 h [14]. Among chronic ventilator-dependent patients, a significant reduction of healthcare infections indicators, such as antibiotic treatment initiation events, fever days and antibiotic usage, was described when the HCWs wore copper-oxide impregnated textiles instead of regular hospital textiles [15]. Copper-impregnated linens were even described to reduce healthcare-associated *C. difficile* infections [16]. Despite all the encouraging results, the permanent exposure of nosocomial pathogens to a biocidal agent is likely to enhance tolerance to this agent [17]. *A.* *baumannii*, for example, has been described to become resistant to copper, also by exposure to subinhibitory concentrations of copper [18]. The increased tolerance may well be explained by copper efflux systems [19]. Certain *P. aeruginosa* isolates have also been found to possess copper tolerance [20]. Items with permanent biocidal impregnation should therefore be regarded with great caution, because it seems to be a matter of time before nosocomial pathogens develop a tolerance to them, possibly even a cross-tolerance to other biocidal agents or antibiotics [21], [22], [23].

## Conclusions

Contaminated textiles or fabrics may be a source of transmission for weeks. The presence of pathogens on the coats of healthcare workers is associated with the presence of pathogens on their hands, demonstrating the relevance of textile contamination in patient care.

## Notes

### Competing interests

The author declares that he has no competing interests.

## Figures and Tables

**Table 1:**
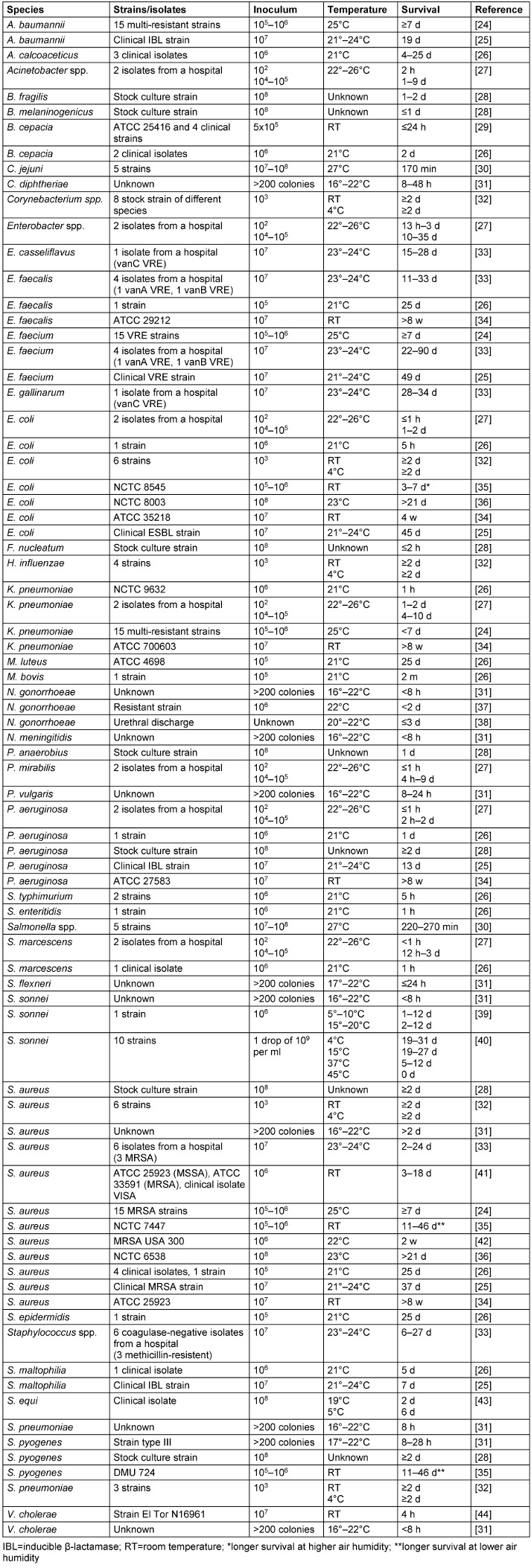
Survival of bacteria on cotton

**Table 2:**
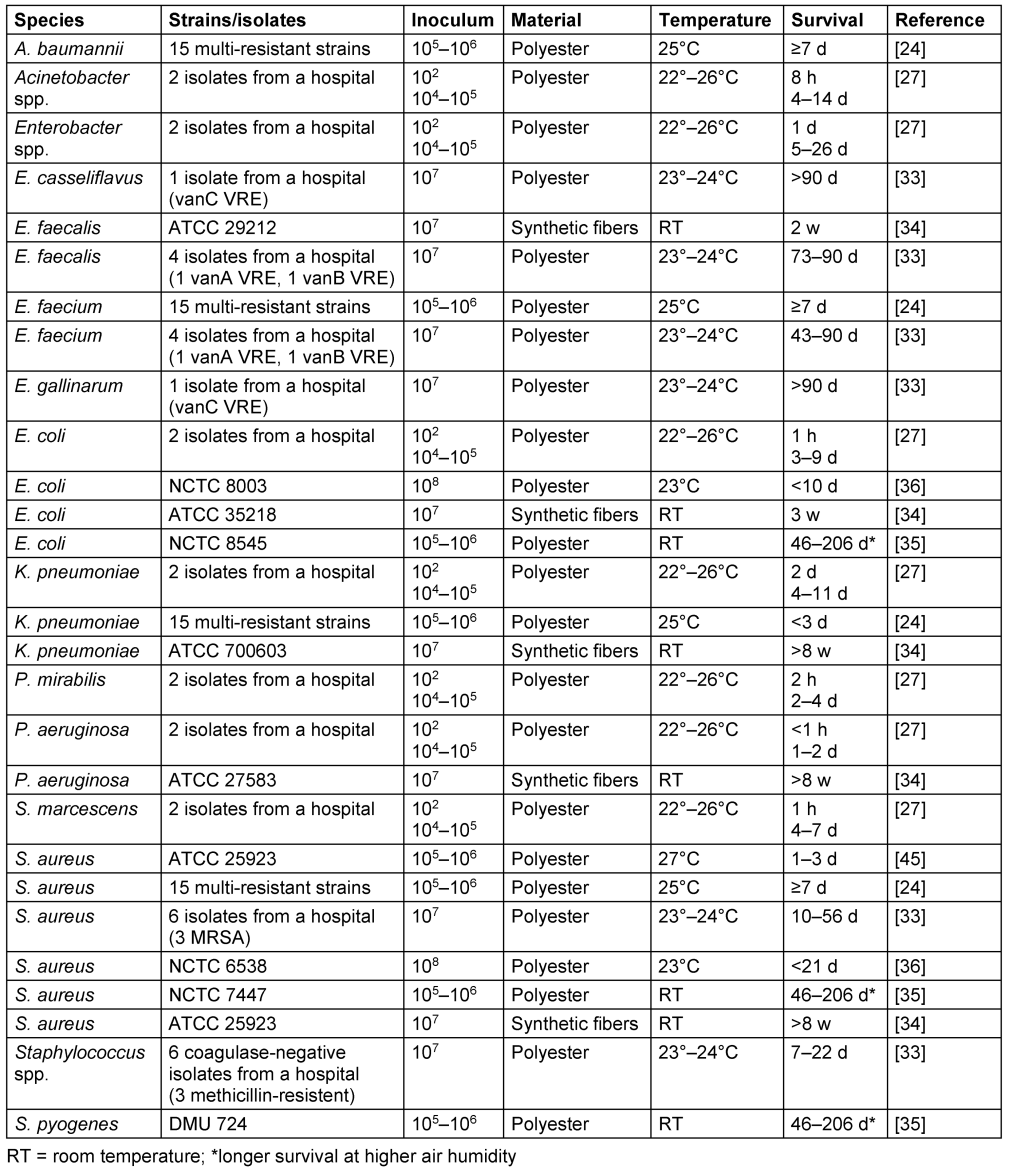
Survival of bacteria on synthetic fibers

**Table 3:**
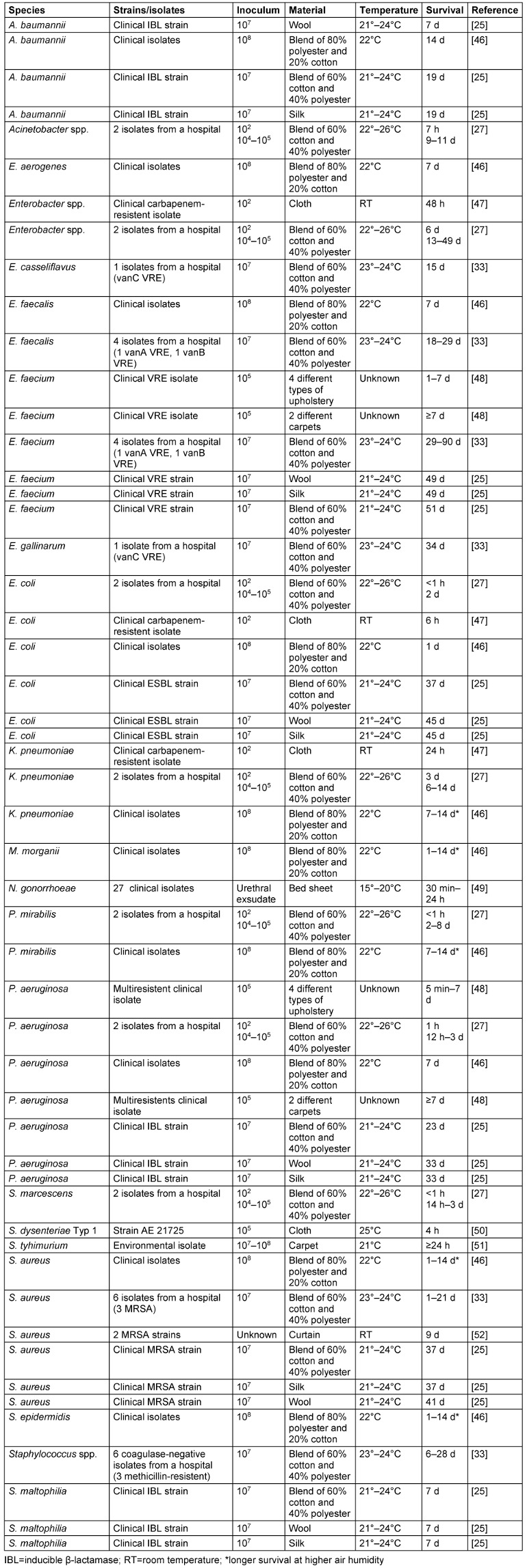
Survival of bacteria on mixed and other fibers

**Table 4:**
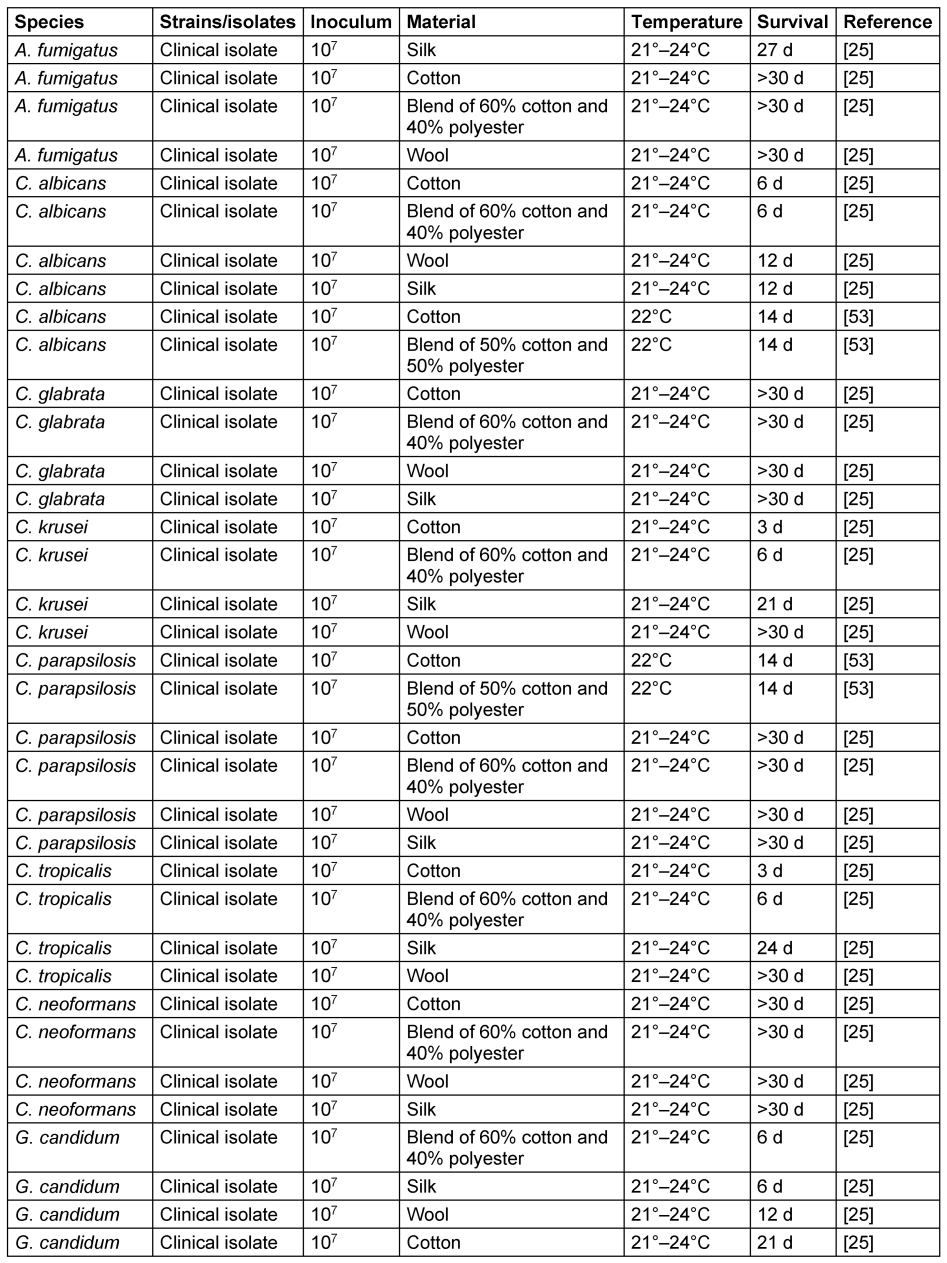
Survival of fungi on different types of fibers

**Table 5:**
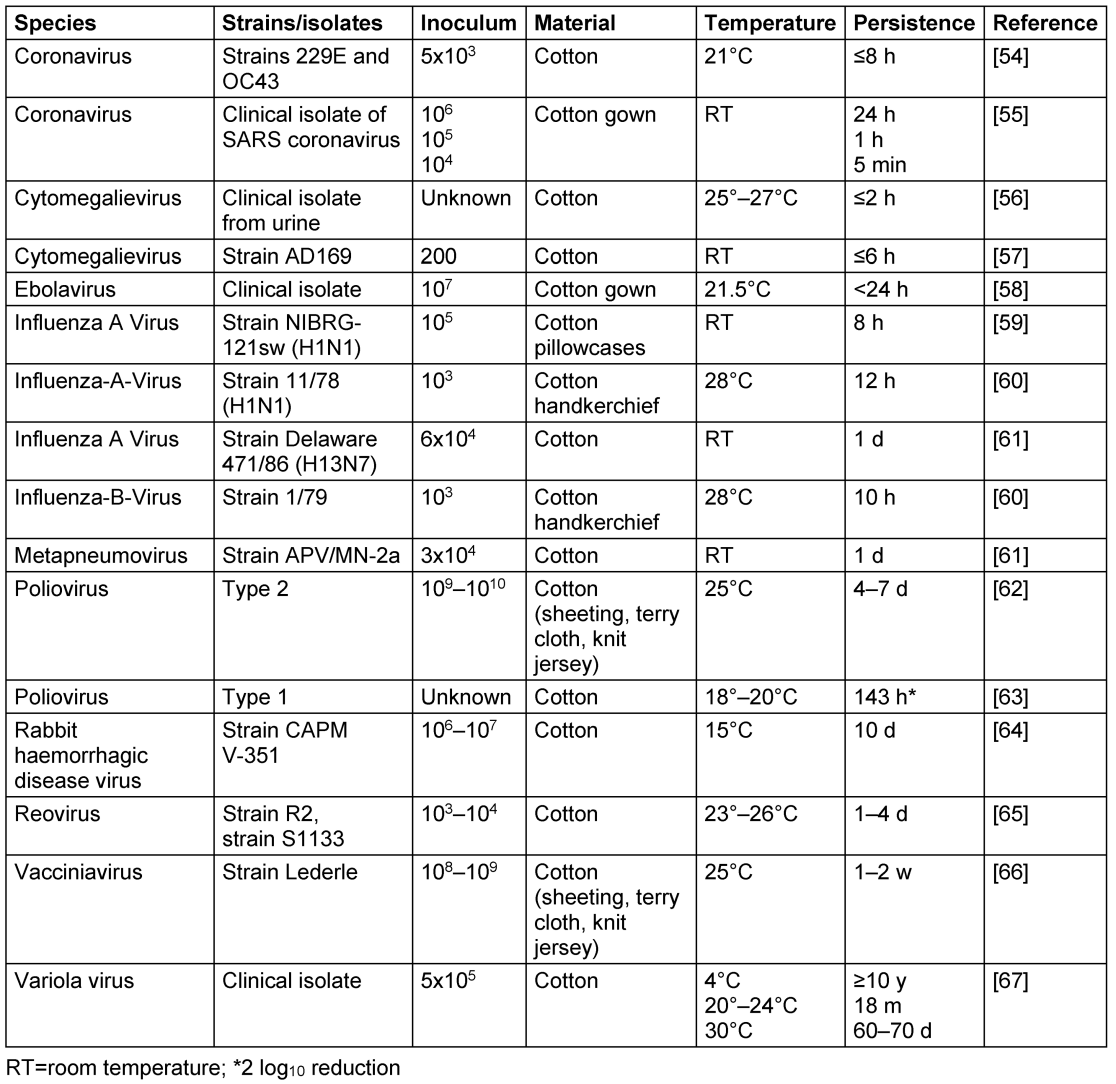
Persistence of viruses on cotton

**Table 6:**
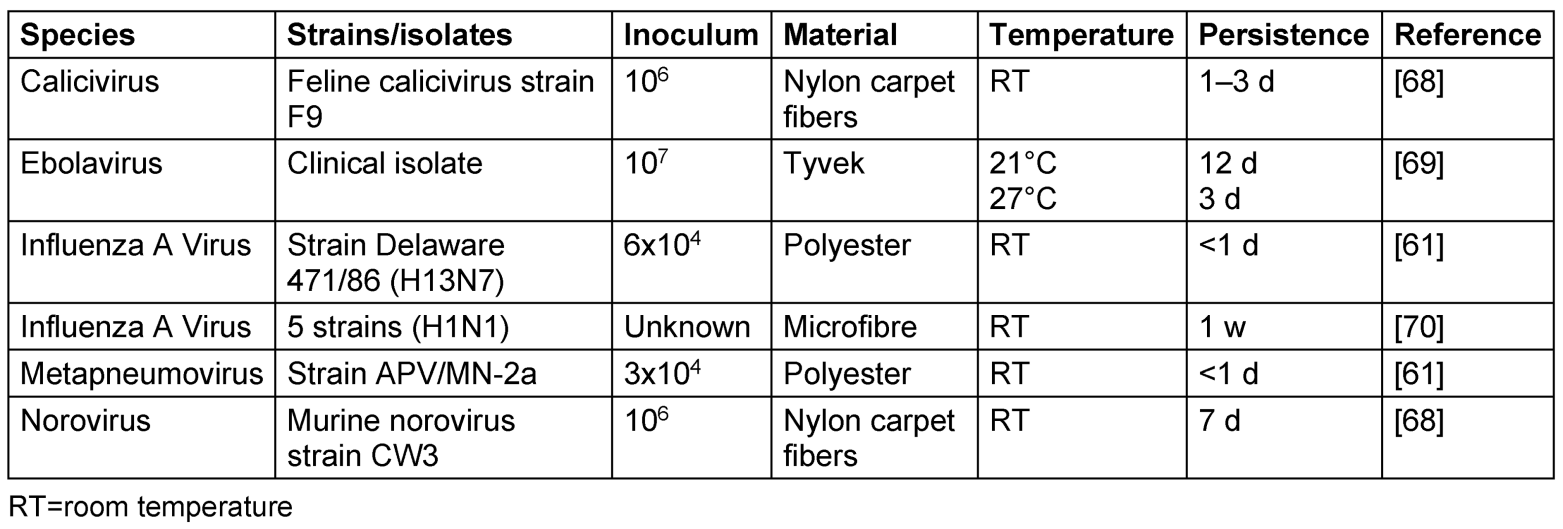
Persistence of viruses on synthetic fibers

**Table 7:**
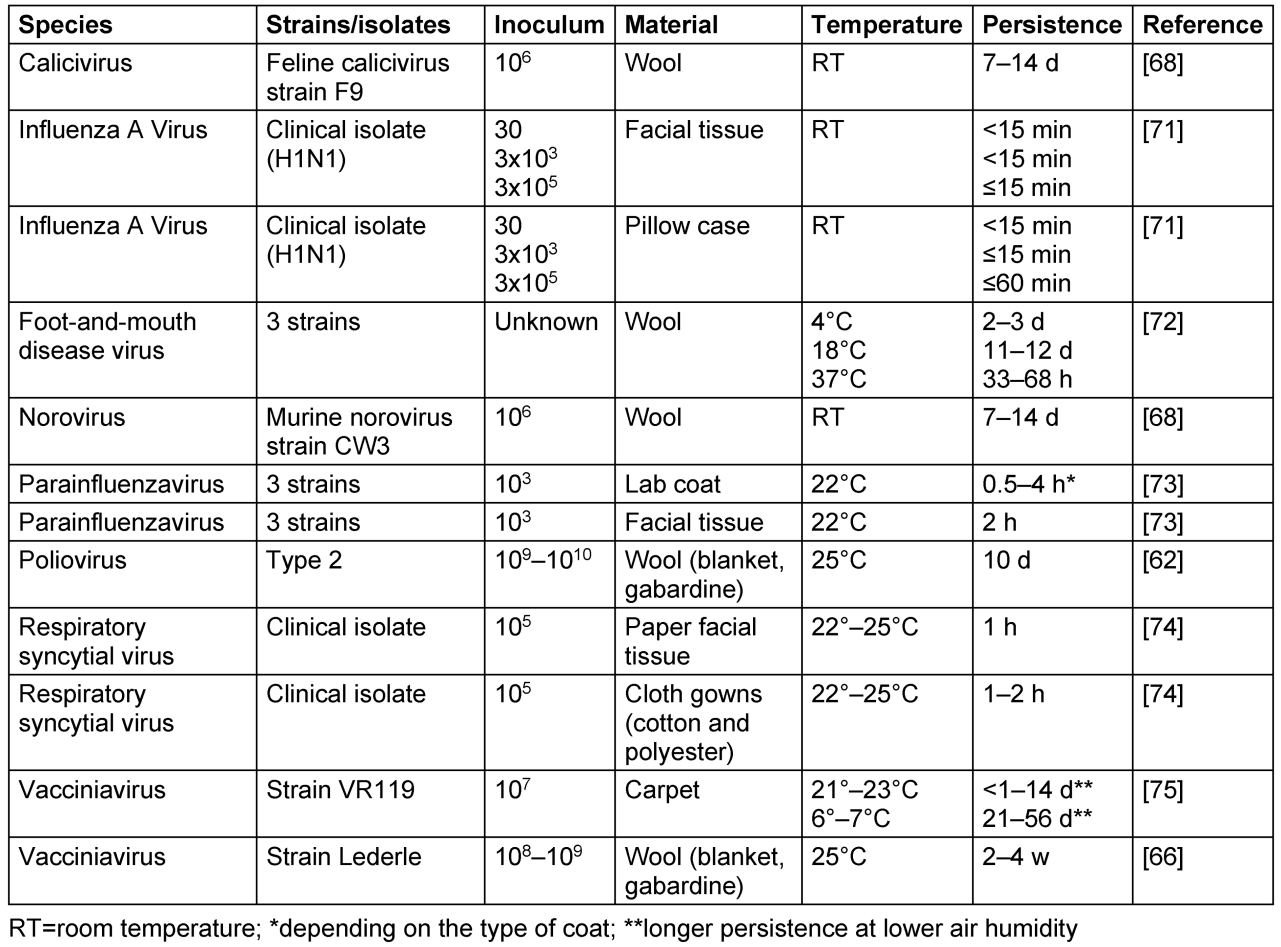
Persistence of viruses on mixed and other fibers
